# Rapid Characterization of Components in *Bolbostemma paniculatum* by UPLC/LTQ-Orbitrap MS^n^ Analysis and Multivariate Statistical Analysis for Herb Discrimination

**DOI:** 10.3390/molecules23051155

**Published:** 2018-05-11

**Authors:** Yanling Zeng, Yang Lu, Zhao Chen, Jiawei Tan, Jie Bai, Pengyue Li, Zhixin Wang, Shouying Du

**Affiliations:** School of Chinese Materia Medica, Beijing University of Chinese Medicine, Yangguang South Avenue, Fangshan District, Beijing 102488, China; zengyl@bucm.edu.cn (Y.Z.); landocean28@163.com (Y.L.); zhaochen223713@126.com (Z.C.); 18798830407@163.com (J.T.); baijie22811@163.com (J.B.); pengyuelee@126.com (P.L.)

**Keywords:** *Bolbostemma paniculatum*, identification, LTQ-Orbitrap, UPLC, multivariate statistical analysis

## Abstract

*Bolbostemma paniculatum* is a traditional Chinese medicine (TCM) showed various therapeutic effects. Owing to its complex chemical composition, few investigations have acquired a comprehensive cognition for the chemical profiles of this herb and explicated the differences between samples collected from different places. In this study, a strategy based on UPLC tandem LTQ-Orbitrap MS^n^ was established for characterizing chemical components of *B. paniculatum*. Through a systematic identification strategy, a total of 60 components in *B. paniculatum* were rapidly separated in 30 min and identified. Then based on peak intensities of all the characterized components, principle component analysis (PCA) and hierarchical cluster analysis (HCA) were employed to classify 18 batches of *B. paniculatum* into four groups, which were highly consistent with the four climate types of their original places. And five compounds were finally screened out as chemical markers to discriminate the internal quality of *B. paniculatum*. As the first study to systematically characterize the chemical components of *B. paniculatum* by UPLC-MS^n^, the above results could offer essential data for its pharmacological research. And the current strategy could provide useful reference for future investigations on discovery of important chemical constituents in TCM, as well as establishment of quality control and evaluation method.

## 1. Introduction

*Bolbostemma paniculatum* (Maxim.) Franquet is a traditional Chinese medicine (TCM), the bulb of which has been often used to treat various diseases [1]. For decades, plenty of attentions of modern pharmaceutical studies have being attracted by *B. paniculatum* because of its potential activity of anti-tumor, anti-viral, anti-inflammatory and detoxication, etc. [2]. Hitherto, more than 70 different compounds have been separated and identified from this herb, and they are attributed to seven categories including alkaloids, flavonols, sterols, triterpenoid saponins, anthraquinones, tetracyclic triterpenoids and others. The chemical constitutes of *B. paniculatum* is so complex that few investigations have got a comprehensive cognition for the chemical profiles of this herb and explicated the differences between samples collected from different places. And it is a fact that *B. paniculatum* is widely cultivated in many provinces in China, such as Shaanxi, Gansu, Shanxi, Ningxia, Shangdong, Henan and Yunnan. The natural conditions such as temperature, humidity and soil in these original places are different, which leads to the obvious quality differentiation of harvested herbs. Li, X.J. et al. [3] established the fingerprints of *B. paniculatum* of 15 different original places by HPLC, using multiple chromatographic peaks in the fingerprints to characterize the overall chemical composition of the herbs and dividing the samples into three categories by cluster analysis. This investigation indicates that there are some differences in the internal quality of *B. paniculatum* from different original places, but due to the lack of subsequent peaks identification process, it cannot point out the specific components which cause those differences. Thus, it is very necessary and challenging to establish an efficient strategy to comprehensively characterize the chemical components of *B. paniculatum*, and to discover several characteristic markers which can be applied to discriminating the herbs from different original places and control their qualities.

In last several years, based on the efficient and fast separation performance of UPLC, as well as the high sensitivity of MS, UPLC tandem MS has turned into an important technology for characterization of chemical components in TCM [4,5]. Especially, UPLC coupled with hybrid LTQ-Orbitrap MS system is applied by more and more researchers in this field [6,7,8], because it is ideal for the identification of natural compounds by obtaining accurate molecular mass and multistage MS^n^ fragment ions of samples to be tested. The LTQ-Orbitrap instrument consists of a two dimensional linear ion trap and an Orbitrap, allowing two different types of scan modes independently and synchronously [9]. In this study, a strategy based on UPLC combined with LTQ-Orbitrap MS^n^ was established for characterizing various chemical components in *B. paniculatum*. Through the summarized MS^n^ fragmentation patterns of reference compounds and systematic identification strategy, a total of 60 components belonged to those seven reported compound types in *B. paniculatum* were rapidly isolated in 30 min and identified for the first time. On the basis of the peak intensities of all the characterized components, multivariate statistical analysis methods including principle component analysis (PCA) and hierarchical cluster analysis (HCA) were employed to classify the bulbs of *B. paniculatum* from different original places. As results, 18 batches of herbs could be unambiguously clustered into four groups, which were consistent with the four climate types of their original places very well. Moreover, five compounds were finally screened out as the important chemical markers to discriminate the internal quality of *B. paniculatum*. For all we know, this is the first study to systematically establish the chemical composition profile of *B. paniculatum* by UPLC-MS^n^ analysis, the results of which could provide essential data for its pharmacological research. The current strategy could be followed by future researches on the identification and discovery of key chemical constituents in TCM, and offer useful reference for the establishment of quality control and evaluation method.

## 2. Results and Discussion

### 2.1. Optimization of UPLC and LTQ-Orbitrap MS^n^ Conditions

For the sake of acquiring chromatograms with intense peak response and resolution, mobile phase compositions were firstly optimized. Compared with methanol/water, the acetonitrile/water system showed higher baseline stability and lower pressure, as well as stronger elutive and isolative ability for investigated components. When bits of formic acid was added into the water phase, the shapes of most peaks were improved apparently. Thus, it was finally decided that acetonitrile/0.1% formic acid aqueous solution was used as the mobile phase. After optimizing the gradient elution program, the column temperature was set at 40 °C to reduce the pressure, and flow rate was constant at 0.3 mL/min.

To acquire high sensitivity for most analytes, some parameters of heated electrospray ionization (HESI) source were also optimized by multiple experiments, including sheath gas flow, auxiliary gas flow, spray voltage, source heater temperature, capillary temperature, capillary voltage and tube lens voltage. These parameters contributed little directly to total ion current chromatogram (TIC) but was extremely crucial for MS^n^ fragmention. The optimal conditions were set as follows: sheath gas flow, 40 arb; auxiliary gas flow, 20 arb; spray voltage, 4 kV/3 kV (positive/negative ESI mode); source heater temperature, 300 °C; capillary temperature, 350 °C; capillary voltage, 25 V/35 V (positive/negative ESI mode); tube lens voltage, 110 V. Because the molecular mass of all known compounds in *B. paniculatum* was distributed in the range of 100–1600 Da, in full scan mode the mass spectra were acquired in the *m*/*z* range of 50–1600 Da, and the resolution was empirically set as 100,000. Moreover, the size of collision-induced dissociation (CID) energy were also considered. The optimal energy for MS^2^ and MS^3^ were 30 V and 35 V, under which fragment ions with appropriate mass could be prodeved at the resolution of 60,000 and 30,000, respectively.

### 2.2. UPLC/LTQ-Orbitrap MS^n^ Analysis of B. paniculatum

**Figure 1:**
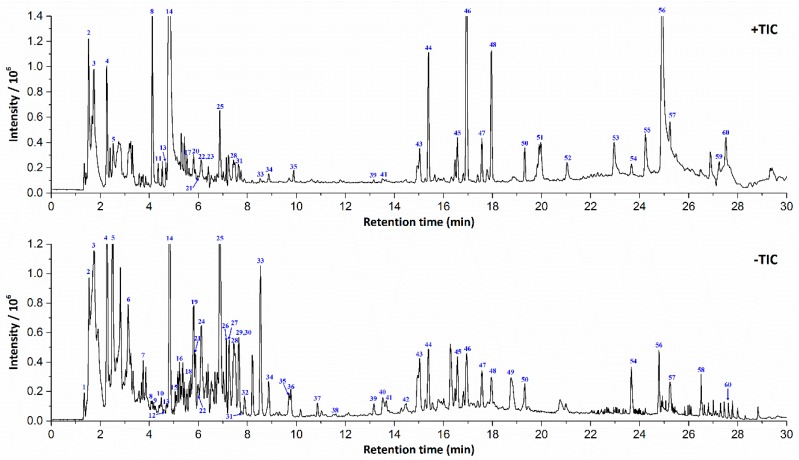
The representative total ion current chromatogram (TIC) of *Bolbostemma paniculatum* extracts in positive and negative ESI modes.

The optimized UPLC/LTQ-Orbitrap MS^n^ conditions were applied for characterization of chemical components in *B. paniculatum* extracts. The TIC in positive and negative ESI modes was shown in Figure 1. We attributed all the reported compounds in *B. paniculatum* into seven types on the grounds of their chemical structures: alkaloids, flavonols, sterols, triterpenoid saponins, anthraquinones, tetracyclic triterpenoids and others. Except sterols, most compounds showed strong response and typical fragmentation in the negative ESI mode, especially the flavonols, triterpenoid saponins, anthraquinones and polyphenols, which contain phenolic hydroxyl group in chemical structure. Thus, the targeted MS^n^ experiments for β-sitosterol (**5**) were conducted in positive mode, and others were in negative mode. The identification of components in *B. paniculatum* was performed based on an established systematic strategy [8]. First of all, the chemical elemental composition for each targeted peak was deduced by the accurate mass spectra of designated deprotonated/protonated molecular ions or adduct ions using a formula predictor, as well as their corresponding isobaric molecular ions. The proposed molecular formulas were also approved by additional judgements such as nitrogen rule, elemental composition of fragment ions and general formula features of natural compounds. Then the formulas were searched in self-built chemical database of *B. paniculatum* to match the known structures. For those formulas not included in the self-built database, they could be input into the SciFinder database for screening potential compounds, and the hits was refined in the genus of *Bolbostemma*. The next process was to verify components after learning the knowledge of characteristic product ions and fragmentation rules of various types of compounds, and the MS^n^ fragmentation patterns of eight reference compounds were sufficiently investigated (Table 1). Those components had the identical retention time, mass and fragment ions with the reference compounds were firstly identified undoubtedly. Other components could be identified via comparing the fragmentation patterns with those known analogous compounds and referring reported structures in literatures. Finally, a total of 60 compounds in *B. paniculatum* extracts were identified or tentatively identified. The retention time, *m*/*z* values of adduct ions and MS^n^ fragment ions in positive/negative ESI modes, mass error, accurate molecular mass, formula and confidence levels of identity [10] of all the identified compounds were completely summarized in Table S1.

### 2.3. Structural Charscterization and Identification of Various Types of Components in B. paniculatum

#### 2.3.1. Structural Characterization and Identification of Alkaloids

One of the representative alkaloids adenosine (**1**, peak 5) [11] were selected as reference compounds to investigate the MS^n^ fragmentation patterns of alkaloids in *B. paniculatum* (see Figure S1). The deprotonated molecular ion *m/z* 266 [M − H]^−^ of adenosine could be easily formed in negative ESI mode, and then it dehydrated (losing H_2_O) to form [M − 18 − H]^−^ MS^2^ fragment ion of *m/z* 248. Fragment ion *m/z* 238 [M − 28 − H]^−^ was produced from *m/z* 266 opening C-ring and decarbonylation (losing CO) at C_4_. Fragment *m/z* 222 [M − 44 − H]^−^ was also formed from *m/z* 266 via opening C-ring and losing one ethenol (C_2_H_4_O), and its further cleavage of entire C-ring formed the fragment ion *m/z* 134 [M − 132 − H]^−^. In this way, other four alkaloids were identified according to their molecular mass, formula, MS^n^ fragments and related literatures, including 2-acetylpyrrole (peak 2) [12], 4-(2-formyl-5-methoxymethylpyrrol-1-yl)butyric acid methyl ester (peak 11) [12], 9-octadecenamide (peak 56) [13] and (E)-*N*-hydroxy phenyl ethyl-3-(4-hy-droxy-3-methoxy phenyl) acrylamide (peak 57) [14]. Among them, 9-octadecenamide was identified from the genus of *Bolbostemma* for the first time. Its protonated molecular ion was *m/z* 282 in positive ESI mode, which could easily lose one formamide (CH_3_NO) to form the only MS^2^ fragmentation ion *m/z* 237 [M − 45 + H]^+^.

#### 2.3.2. Structural Characterization and Identification of Flavonols

As shown in Figure S2, the deprotonated molecular ion of quercitrin (**3**, peak 14) [15] was observed at *m/z* 447 [M − H]^−^ in negative ESI mode, which could easily yield the MS^2^ fragment ion *m/z* 419 [M − 28 − H]^−^ after decarbonylation, or yield the fragment ion *m/z* 327 [M − 120 − H]^−^ after losing 4-methyl-1,2-benzenediol (C_7_H_8_O_2_). Moreover, the deprotonated molecular ion *m/z* 447 could form the fragment ion *m/z* 301 [M − 146 − H]^−^, which was by losing one 2-hydroxy-d-glucal (C_6_H_10_O_4_) from the side chain. Another fragment ion of deprotonated molecular ion *m/z* 447 was *m/z* 284 [M − 163 − H]^−^, which was formed by homolytic cleavage of glycosidic linkage losing one rhamnose radical (C_6_H_11_O_5_) from parent ion. The fragment ion *m/z* 301 could form the fragment *m/z* 257 [M − 190 − H]^−^ by decarboxylation (losing CO_2_) at A-ring. The MS^3^ fragment ion *m/z* 179 [M − 268 − H]^−^ was derived from losing pyrocatechol (C_6_H_6_O_2_) from C-ring and dehydration, and its further decarbonylation formed the fragment ion *m/z* 151 [M − 296 − H]^−^. In the samilar way, other two flavonols were identified as 3-*O*-[β-d-pyranrham-nose-(1-6)-β-d-galactopyranose]-5,7,4′-trihydroxyl flavone (peak 12) [16,17] and quercetin-3-*O*-α-l-arabinopyranoside (peak 16) [17], respectively.

#### 2.3.3. Structural Characterization and Identification of Sterols

A typical sterol, β-sitosterol (**5**, peak 17) [18] was taken as an example to investigate the MS^n^ fragmentation pattern of sterols in *B. paniculatum* (see Figure S3). The protonated molecular ion of β-sitosterol was *m/z* 415 [M + H]^+^ in positive ESI mode, and its intramolecular dehydration between C_1_-OH and C_2_-H at A-ring produced the MS^2^ fragment ion *m/z* 397 [M − 18 + H]^+^. As a result of electron receptor effect of the large four-rings conjugate system, the single bond between C_19_ and C_20_ was unstable, and the fragment ion *m/z* 397 could easily lose a 3-ethyl-2-methylheptane (C_10_H_22_) from the side chain producing MS^3^ fragment ion *m/z* 255 [M − 160 + H]^+^. Then the D-ring in fragment *m/z* 255 opened and one propane (C_3_H_8_) was lost to generate the fragment ion *m/z* 215 [M − 200 + H]^+^. Another fragmentation way of protonated molecular ion *m/z* 415 was that it firstly lost a 3-ethyl-2-methylheptane to yield the fragment *m/z* 273 [M − 142 + H]^+^, and then lost one propane (C_3_H_8_) to yield the fragment *m/z* 233 [M − 182 + H]^+^. At last, the fragment *m/z* 233 dehydrated at C_1_ to generate the fragment ion *m/z* 215 [M − 200 + H]^+^. In this way, other 14 sterols were identified or tentatively identified, including stigmasta-7,16,25-triene-3-ol (peak 20) [19], stigmasta-7,22,25-triene-3-ol (peak 23) [20,21], uzarigenin-3-β-sophoroside (peak 43) [22], sileneoside H (peak 45) [23], daucosterol (peak 46) [20], frugoside (peak 47) [24], stigmasta-7,22,25-triene-3-*O*-β-d-glucopyranoside (peak 48) [20,21], integristerone A-25-acetate (peak 50) [25], 24(28)-dehydromakisterone A (peak 51) [26], β-sitosterol palmitate (peak 53) [18], stigmasta-7,22,25-triene-3-*O*-β-d-(6′-palmitoyl) glucopyranoside (peak 54) [27], stigmasta-7,22,25-triene-3-*O*-nonadecanoic acid ester (peak 55) [21,27], (3β,22E)-stigmasta-7,22,25-trien-3-yl-β-d-glucopyranoside (peak 59) [20,21] and 3-oxo-androsta-1,4-dien-17a′-spiro-2′-3′-oxo-oxetane (peak 60) [28], respectively. Among them, uzarigenin-3-β-sophoroside, sileneoside H, frugoside, integristerone A-25-acetate, 24(28)-dehydromakisterone A and 3-oxo-androsta-1,4-dien-17a′-spiro-2′-3′-oxo-oxetane were identified from the genus of *Bolbostemma* for the first time.

#### 2.3.4. Structural Characterization and Identification of Triterpenoid Saponins

A total of 20 triterpenoid saponins were identified from the extracts of *B. paniculatum*. Herein, tubeimoside I was taken as an example to elucidate the mass fragmentation pattern of this type of components. As shown in Figure S4, the fragmentation of deprotonated molecular ion *m/z* 1317 [M − H]^−^ of tubeimoside I (**6**, peak 27) [29] firstly occurred at the five-carbons linkage of the large ring. The ring was broken, and then lost one 3-hydroxybutanoic acid residue (C_4_H_4_O_2_) to produce the MS^2^ fragment ion *m/z* 1233 [M − 84 − H]^−^, followed by the second neutral loss of one acetic acid (C_2_H_4_O_2_) to produce the fragment ion *m/z* 1173 [M − 144 − H]^−^. Due to the existence of multiple glycosyls, the next fragmentation step was to lose monosaccharide residues one by one, and the corresponding products included fragment ions *m/z* 781 and *m/z* 649, et al. In this way, other 19 triterpenoid saponins were identified or tentatively identified, including tubeimoside II (peak 18) [29], tubeimoside IV (peak 19) [30], actinostemmoside F (peak 21) [31], 7β,18,20,26-tetrahydroxy-(20S)-dammar-24E-en-3-*O*-α-l-(3-acetyl)arabinopyranosyl-(1→2)-β-d-glucopyranoside (peak 22) [32], 7β,18,20,26-tetrahydroxy-(20S)-dammar-24E-en-3-*O*-α-l-arabinopyranosyl-(1→2)-β-d-(6-acetyl)-glucopyranoside (peak 24) [32], lobatoside D (peak 25) [33], tubeimoside III (peak 26) [29], lobatoside B (peak 28) [33], dexylosyltubeimoside III (peak 29) [34], lobatoside C (peak 30) [30], lobatoside G (peak 31) [33], tubeimoside V (peak 32) [29], lobatoside F (peak 33) [33], actinostemmoside H (peak 34) [31], 7β,20,26-trihydroxy-(20S)-dammar-24E-en-3-*O*-α-l-arabinopyranosyl-(1→2)-β-d-glucopyranoside (peak 35) [32], lobatoside A (peak 36) [35], 7β,20,26-trihydroxy-(20S)-dammar-24E-en-3-*O*-α-l-(3-acetyl)arabinopyranosyl-(1→2)-β-d-glucopyranoside (peak 37) [32], actinostemmoside E (peak 38) [31] and 3-*O*-α-l-arabinopyranosyl(1→2)-β-d-glucopyranosyl-bayogenin-28-*O*-β-d-xylopyranosyl(1→3)-α-l-rhamnopyranosyl(1→2)-α-l-arabinopyranoside (peak 49) [36].

#### 2.3.5. Structural Characterization and Identification of Anthraquinones

Two anthraquinones were identified from the extracts of *B. paniculatum*, including emodin (**7**, peak 39) [20,21] and emodinmonomethylether (peak 41) [37]. As shown in Figure S5, in negative ESI mode, the deprotonated molecular ion *m/z* 269 [M − H]^−^ of emodin was easy to decarboxylated from the A-ring and B-ring, forming the MS^2^ fragment ion *m/z* 225 [M − 44 − H]^−^. Then the fragment *m/z* 225 furtherly decarbonylated to form smaller fragment ion *m/z* 197 [M − 72 − H]^−^. The fragment ion *m/z* 241 [M − 28 − H]^−^ was derived from the direct decarboxylation at A-ring of deprotonated molecular ion *m/z* 269. As the methylated derivative of emodin, the fragmentation pattern of deprotonated molecular ion *m/z* 283 [M − H]^−^ of emodinmonomethylether was completely same to emodin, except for the first process losing one methyl to produce the fragment ion *m/z* 269 [M − 15 − H]^−^.

#### 2.3.6. Structural Characterization and Identification of Tetracyclic Triterpenoids and Other Components

As depicted in Figure S6, in negative ESI mode, the fragmentation process of deprotonated molecular ion *m/z* 557 [M − H]^−^ of cucurbitacin B (**8**, peak 44) [21] started from the loss of acetic acid (C_2_H_4_O_2_) at side chain to yield the MS^2^ fragment ion *m/z* 515 [M − 42 − H]^−^. Due to the multiple hydroxyls substitution, the fragment ion *m/z* 515 then showed sequential dehydrations in the MS^n^ experiment, i.e., *m/z* 497, *m/z* 479, *m/z* 461 and *m/z* 443 fragment ions. Certainly, the deprotonated molecular ion *m/z* 557 could also dehydrate firstly to yield the fragment ion *m/z* 539 [M − 18 − H]^−^. In this way, other two tetracyclic triterpenoids were identified as 23,24-dihydroisocucurbitacin B (peak 40) [30] and cucurbitacin E (peak 42) [21,30], respectively.

As the typical representatives of other components, the fragmentation pattern of chlorogenic acid (**2**, peak 10) [16] and scopoletin (**4**, peak 15) [16] in negative ESI mode were firstly investigated, and their MS^n^ spectra and proposed fragment ions was shown in Figures S7 and S8, respectively. In negative ESI mode, the deprotonated molecular ion *m/z* 353 [M − H]^−^ of chlorogenic acid could dehydrate through hydroxyl at C_1_ and adjacent hydrogen to form MS^2^ fragment ion *m/z* 335 [M − 18 − H]^−^, or lose one CO_2_ from carboxyl at C_1_ to form fragment ion *m/z* 309 [M − 44 − H]^−^. The fragment ion *m/z* 191 [M − 162 − H]^−^ of high abundance came from the easy cleavage of entire B-ring of deprotonated molecular ion *m/z* 353. The further fragmentation of precursor ion *m/z* 191 was orderly to dehydrate, lose one formic acid (CH_2_O_2_) and open A-ring to yield MS^3^ fragment ions *m/z* 173 [M − 180 − H]^−^, *m/z* 127[M − 226 − H]^−^ and *m/z* 85 [M − 268 − H]^−^, respectively. Another polyphenol catechin (peak 6) [16] was also identified in this way. Compared with polyphenols, the MS^n^ fragmentation pattern of coumarins in negative ESI mode was more understandable. The deprotonated molecular ion of scopoletin was *m/z* 191 [M − H]^−^. It could lose one methyl radical through homolytic cleavage to produce MS^2^ fragment ion *m/z* 176 [M − 15 − H]^−^. The MS^3^ fragment ion *m/z* 148 [M − 43 − H]^−^ came from decarbonylation of *m/z* 176, and further decarboxylation of *m/z* 176 produced *m/z* 104 [M − 87 − H]^−^. Also, decarbonylation of *m/z* 148 produced fragment ion *m/z* 120 [M − 71 − H]^−^. According to the molecular mass, formulas, MS^n^ fragment ions and related literatures, nine other components including sucrose (peak 1) [38], stachyose (peak 3) [39], α-hydroxyacetone glucoside (peak 4) [20], maltol (peak 7) [18,20,21], pyrogallol (peak 8) [40], n-butyl-β-d-fructopy ranoside (peak 9) [18], 5-*O*-feruloylquinic acid (peak 13) [14], di-butyl phthalate (peak 52) [14] and hexadecanoic acid (peak 58) [11] were also identified or tentatively identified, respectively (Table S1). The MS^n^ fragmentation pathways of these compounds were relative simple, so they were not described detailedly here. Among them, pyrogallol was identified from the genus of *Bolbostemma* for the first time, and stachyose was identified from *B. paniculatum* for the first time.

### 2.4. Multivariate Statistical Analysis with PCA and HCA

**Figure 2:**
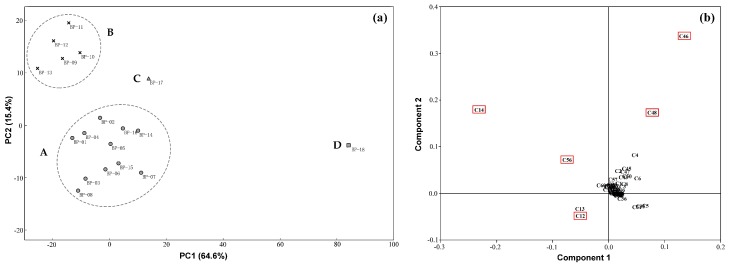
PCA score plot (**a**) and correlation plot (**b**) of 18 batches of *Bolbostemma paniculatum* based on all the characterized components.

**Figure 3:**
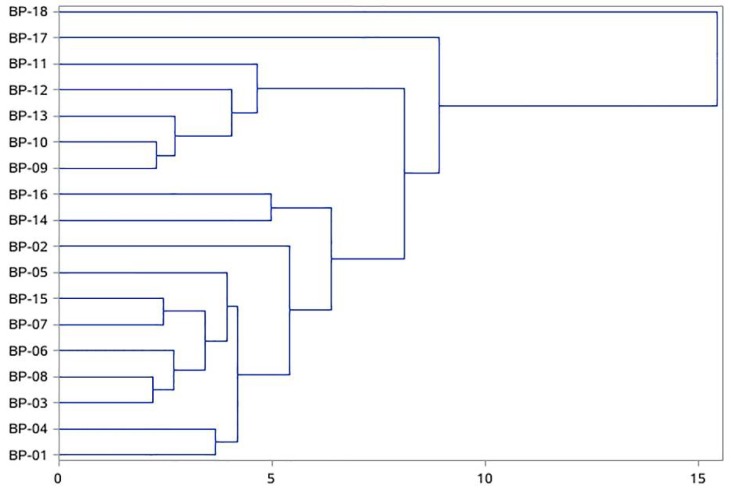
Dendrograms of hierarchical cluster analysis (HCA) for 18 batches of *Bolbostemma paniculatum* based on the five investigated markers.

In order to discriminate the differences among the 18 batches of *B. paniculatum* collected from different places, PCA multivariate statistical analysis on the basis of the chromatographic profiling for all the characterized components was initially adopted. As results, eighty percent of the whole variances were explained by the principal factorial plane, in which PC1 and PC2 hold 64.6% and 15.4%, respectively. As shown in Figure 2a, 18 batches of herbs could be unambiguously clustered into four groups. Interestingly, these four groups were consistent with the four kinds of climate types (shown in Table 2) of the original place of these herbs very well, including temperate (continental) monsoon climate (group A), warm temperate semi-humid (continental) monsoon climate (group B), warm temperate-subtropical monsoon climate (group C) and subtropical monsoon climate (group D). Obviously, the score plot of BP-18 was far away from the other three groups on account of its original place was Yunnan, which locates in the zone of subtropical monsoon climate. And it was the hot and humid climate that led to the great differences in chemical composition of BP18 compared with other herbs. Analogously, BP-17 lay between the group A and B, because its original place Henan is the boundary between warm temperate and subtropical monsoon climate of China. On the other hand, according to the correlation plot (Figure 2b), it was found that five components including 3-*O*-[β-d-pyranrham-nose-(1-6)-β-d-galactopyranose]-5,7,4′-trihydroxyl flavone (peak 12), quercitrin (peak 14), daucosterol (peak 46), stigmasta-7,22,25-triene-3-*O*-β-d-glucopyranoside (peak 48) and 9-octadecenamide (peak 56) contributed most to the grouping result, which were much more statistically significant in chemotaxonomy than the other identified components. In addition, the result of HCA based on the five predicted chemical markers proved that the differences in chemical composition is obvious among the four different groups of herbs (see Figure 3), which further confirmed the calculated results of PCA. Therefore, these five common components could be regarded as the most important chemical markers to discriminate the internal quality of *B. paniculatum*. However, owing to the limitation of the number of herb samples collected, the results of multivariate statistical analysis here were not systematic. The further investigation would be carried out in the future.

## 3. Materials and Methods

### 3.1. Reagents and Materials

Eighteen batches of *B. paniculatum* bulbs were collected from seven provinces of China (Table 2). All the herb samples were authenticated with morphological and histological methods by Prof. Yuan Zhang (School of Chinese Materia Medica, Beijing University of Chinese Medicine). Voucher specimens were preserved at the authors’ laboratory.

The reference compounds adenosine (**1**), chlorogenic acid (**2**), quercitrin (**3**), β-sitosterol (**5**), emodin (**7**) and scopoletin (**8**) were purchased from Shanghai Yuanye Biological Technology Co., Ltd. (Shanghai, China); tubeimoside I (**4**) was from National Institutes for Food and Drug Control (Beijing, China); cucurbitacin B (**8**) was from Shanghai Standard Technology Co., Ltd. (Shanghai, China).

Acetonitrile, methanol and formic acid (all MS grade) were purchased from Sigma-Aldrich (St. Louis, MO, USA). The ultra-pure water was prepared with the Millipore-Q water purification system (Bedford, MA, USA).

### 3.2. Samples Preparation

After accurately weighed, grounded and sieved through a 65 meshes sieve, 1.0 g air-dried bulbs of *B. paniculatum* were extracted with 20 mL methanol in a 50 mL erlenmeyer flask by ultrasonic extraction for 30 min. After cooling down, the lost volume of methanol was complemented. Then 5.0 mg of eight reference compounds were dissolved into 5 mL methanol to get eight standard solutions, respectively. Finally, the above herb extracts solution and all standard solutions were filtered through a 0.22 μm membrane as the samples.

### 3.3. UPLC Separation

UPLC separation was carried out on a Thermo Ultimate 3000 UPLC platform (Thermo Fisher Scientific, Waltham, MA, USA) equipped with a multichannel UV detector. The chromatographic column used was an Agilent Zorbax RRHD Eclipse Plus C_18_ (150 × 3.0 mm, 1.8 μm; Agilent, Santa Clara, CA, USA), which conducted in 40 °C. The mobile phase was composed of 0.1% formic acid aqueous solution (A) and acetonitrile (B), and the gradient elution program was as follows: 5–38% B at 0–4 min; 38–48% B at 4–10 min; 48–100% B at 10–26 min; 100% B at 26–30 min. The flow rate was constant at 0.3 mL/min. The injection volume was set at 2 μL.

### 3.4. LTQ-Orbitrap MS^n^ Analysis and Data Processing

The LTQ-Orbitrap Elite mass spectrometer (Thermo Fisher Scientific, Waltham, MA, USA) was coupled to the UPLC by a HESI interface. The specific parameters were set as aforementioned. The mass spectrometer calibration was conducted before each experiment. In the MS^n^ experiments, data-dependent scanning was adopted to trigger multistage fragmentation, which was to select the strongest several parent ions in each scanning point as targeted precursor ions for the further fragmentation: four ions for MS^2^ fragmentation and one ion for MS^3^ fragmentation, respectively. The dynamic exclusion function was utilized to prevent the repetitive ion scans and save the analysis time. The software Xcalibur 4.1 (Thermo Fisher Scientific, Waltham, MA, USA) and Mass Frontier 7.0 (Thermo Fisher Scientific, Waltham, MA, USA) were employed to process the UPLC-MS data. To ensure the reliability of the identification results, those peaks with intensity over 10^5^ in TIC were selected for identification. The formulas of all parent and fragment ions in selected peaks were generated according to their accurate mass using a formula predictor. The maximal mass accuracy error was confined to ±3 ppm. In consideration of the possible elemental compositions of existed compounds in *B. paniculatum*, the number of four types of atoms were limited as follows: C ≤ 100, H ≤ 150, O ≤ 50 and N ≤ 10.

### 3.5. Method Validation and Multivariate Statistical Analysis

The repeatability and precision were evaluated by detecting the eight known reference compounds in three groups of independently prepared herb extract solutions five times in 72 h, respectively. And the corresponding relative standard deviation (RSD) of peak intensities was then taken as the index for method validation. As classical multivariate statistical analysis methods, unsupervised PCA and HCA were performed by SAS 9.4 (SAS Institute Inc., Cary, NC, USA) respectively. to illuminate the variances in chemical composition among 18 batches of herb samples from different original places.

## Figures and Tables

**Table 1:** The UPLC-MS^n^ data of eight representative reference compounds in *Bolbostemma paniculatum*.

Category	Compound Name	t_R_ (min)	Formula	Molecular Mass	Positive ESI Mode			Negative ESI Mode		
					Adduct Ions	Mass Error (ppm)	MS^n^ Fragment Ions	Adduct Ions	Mass Error (ppm)	MS^n^ Fragment Ions
Alkaloid	adenosine (**1**) ^a^	2.45	C_10_H_13_N_5_O_4_	267.0968	268.1042 [M + H]^+^	−0.63	-	266.0889 [M − H]^−^	2.18	MS^2^: 248(50.6), 238(100), 222(63.1), 134(33.8)
Polyphenol	chlorogenic acid (**2**)	4.39	C_16_H_18_O_9_	354.0951	-	-	-	353.0854 [M − H]^−^	−1.32	MS^2^: 335(100), 309(71.2), **191**(86.3) ^b^ MS^3^: 173(100), 127(69.2), 85(23.9)
Flavonol	quercitrin (**3**)	4.81	C_21_H_20_O_11_	448.1006	449.1081 [M + H]^+^	−0.58	-	447.0912 [M − H]^−^895.1896 [2M − H]^−^	−0.97	MS^2^: 419(10.1), 327(24.8), **301**(44.5), 284(100), 257(8.81) MS^3^: 179(100), 151(81.2)
Coumarin	scopoletin (**4**)	5.18	C_10_H_8_O_4_	192.0423	-	-	-	191.0335 [M − H]^−^	−0.41	MS^2^: **176**(100) MS^3^: 148(100), 120(26.7), 104(15.2)
Sterol	β-sitosterol (**5**)	5.53	C_29_H_50_O	414.3862	415.3940 [M + H]^+^	−1.34	MS^2^: **397**(89.2), 273(100), 233(13.5) MS^3^: 255(100), 215(35.1)	-	-	-
Triterpenoid saponins	tubeimoside I (**6**)	7.39	C_63_H_98_O_29_	1318.6194	-	-	-	1317.6083 [M − H]^−^	2.88	MS^2^: 1233(100), 1173(47.3), 781(38.5), 649(11.3)
Anthraquinone	emodin (**7**)	13.19	C_15_H_10_O_5_	270.0528	271.0589 [M + H]^+^	−1.230	-	269.0457 [M − H]^−^	0.77	MS^2^: 241(32.2), 225(100), 197(3.98)
Tetracyclic triterpenoids	cucurbitacin B (**8**)	15.27	C_32_H_46_O_8_	558.3193	581.3100 [M + Na]^+^	−2.60	-	557.3102 [M − H]^−^	2.85	MS^2^: 539(100), 515(30.8), 497(90.1) 479(10.2)

**^a^** The bracketed bold figures showed the serial number of corresponding reference compounds. **^b^** The bold *m*/*z* values and bracketed relative peak intensities showed the targeted MS^2^ fragment ions for further MS^3^ fragmentation.

**Table 2:** The original places of 18 batches of *Bolbostemma paniculatum* bulbs and their corresponding climate types.

Sample	Original Place	Climate Type
BP-01	Chunhua, Xianyang, Shaanxi	temperate monsoon climate
BP-02	Xunyi, Xianyang, Shaanxi	temperate monsoon climate
BP-03	Yongshou, Xianyang, Shaanxi	temperate monsoon climate
BP-04	Fengping, Baoji, Shaanxi	temperate monsoon climate
BP-05	Taibai, Baoji, Shaanxi	temperate monsoon climate
BP-06	Yaozhou, Tongchuan, Shaanxi	temperate continental monsoon climate
BP-07	Baota, Yanan, Shaanxi	temperate continental monsoon climate
BP-08	Huxian, Xi’an, Shaanxi	temperate monsoon climate
BP-09	Shangzhou, Shangluo, Shaanxi	warm temperate semi-humid monsoon climate
BP-10	Shangzhou, Shangluo, Shaanxi	warm temperate semi-humid monsoon climate
BP-11	Yangxian, Hanzhong, Shaanxi	warm temperate semi-humid monsoon climate
BP-12	Chengcheng, Weinan, Shaanxi	warm temperate semi-humid continental monsoon climate
BP-13	Wanrong, Yuncheng, Shanxi	warm temperate semi-humid continental monsoon climate
BP-14	Zhenyuan, Qingyang, Gansu	temperate continental monsoon climate
BP-15	Longde, Guyuan, Ningxia	temperate continental monsoon climate
BP-16	Yiyuan, Zibo, Shandong	temperate monsoon climate
BP-17	Luoyang, Henan	warm temperate-subtropical monsoon climate
BP-18	Baoshan, Yunnan	subtropical monsoon climate

## Supplementary Materials

The following are available online, Figure S1: MS^n^ spectra and proposed fragment ions of adenosine (**1**) in negative ion mode; Figure S2: MS^n^ spectra and proposed fragment ions of quercitrin (**3**) in negative ion mode; Figure S3: MS^n^ spectra and proposed fragment ions of β-sitosterol (**5**) in positive ion mode; Figure S4: MS^n^ spectra and proposed fragment ions of tubeimoside I (**6**) in negative ion mode; Figure S5: MS^n^ spectra and proposed fragment ions of emodin (**7**) in negative ion mode; Figure S6: MS^n^ spectra and proposed fragment ions of cucurbitacin B (**8**) in negative ion mode; Figure S7: MS^n^ spectra and proposed fragment ions of chlorogenic acid (**1**) in negative ion mode; Figure S8: MS^n^ spectra and proposed fragment ions of scopoletin (**4**) in negative ion mode; Table S1: All the identified or tentatively identified components from *Bolbostemma paniculatum* extracts and their UPLC-MS^n^ data.
