# Supplementary material for: Rapid Characterization of Components in Bolbostemma paniculatum by UPLC/LTQ-Orbitrap MSn Analysis and Multivariate Statistical Analysis for Herb Discrimination
Source: Molecules. 2018 May 11;23(5):1155. doi: 10.3390/molecules23051155 (PMC6100040; doi:10.3390/molecules23051155)
Supplement: Supplementary file 1 [file molecules-23-01155-s001.pdf]

Table S1. All the identified or tentatively identified components from *Bolbostemma paniculatum* extracts and their UPLC-MS^n data.

No.	t_R (min)	Positive ESI mode			Negative ESI mode			Molecular mass	Formula	Identification	Confidence level of identity[a]
		Adduct ions	Mass error (ppm)	MS^n fragment ions	Adduct ions	Mass error (ppm)	MS^n fragment ions				
**1**	1.64	-	-	-	341.1092 $[M-H]^-$<br>683.2264 $[2M-H]^-$	-0.68	MS^2: 179(100)	342.1162	$C_{12}H_{22}O_{11}$	sucrose	III
**2**	1.73	110.0602 $[M+H]^+$	-0.72	MS^2: 68(100)	108.0453 $[M-H]^-$	1.73	MS^2: 66(100)	109.0528	C_6H_7NO	2-acetylpyrrole	III
**3**	1.91	667.2277 $[M+H]^+$	2.15	-	665.2128 $[M-H]^-$	2.68	MS^2: 647(3.8), 503(12.7), **341**(100)[b]<br>MS^3: 179(100)	666.2219	$C_{24}H_{42}O_{21}$	stachyose	III
**4**	2.25	237.0966 $[M+H]^+$	1.31	-	235.0821 $[M-H]^-$	1.02	MS^2: 179(100)	236.0896	$C_9H_{16}O_7$	α-hydroxyacetone glucoside	III
**5**	2.45	268.1042 [M+H]+	-0.63	-	266.0889 $[M-H]^-$	2.18	MS^2: 248(50.6), 238(100), 222(63.1), 134(33.8)	267.0968	$C_{10}H_{13}N_5O_4$	adenosine (**1**)[c]	I
**6**	3.13	-	-	-	289.0725 $[M-H]^-$	-2.55	MS^2:179(100), 125(76.5), 109(39.8)	290.0790	$C_{15}H_{14}O_6$	catechin	III
**7**	3.53	-	-	-	125.0243 $[M-H]^-$	1.06	MS^2: 81(100)	126.0317	$C_6H_6O_3$	maltol	III
**8**	4.18	127.0393 $[M+H]^+$	-2.59		125.0242 $[M-H]^-$	1.74	MS^2: 83(100)	126.0317	$C_6H_6O_3$	pyrogallol	III
**9**	4.21	-	-	-	235.1183 $[M-H]^-$	1.55	MS^2: 179(100)	236.1260	$C_{10}H_{20}O_6$	n-butyl-β-D-fructopy ranoside	III
**10**	4.39	-	-	-	353.0854 $[M-H]^-$<br>707.1791 $[2M-H]^-$	-1.32	MS^2: 335(100), 309(71.2), **191**(86.3)<br>MS^3: 173(100), 127(69.2), 85(23.9)	354.0951	$C_{16}H_{18}O_9$	chlorogenic acid (**2**)	I
**11**	4.43	240.1235 $[M+H]^+$	-1.94	MS^2: **212**(100), 140(30.1)<br>MS^3:112(100)	-	-	-	239.1158	$C_{12}H_{17}NO_4$	4-(2-formyl-5-methoxymethylpyrrol-1-yl)butyric acid methyl ester	III
**12**	4.66	-	-	-	595.1292 $[M-H]^-$	2.11	MS^2: 433(100), **285**(85.2)<br>MS^3: 241(100), 161(25.4)	596.1377	$C_{26}H_{28}O_{16}$	3-O-[β-D-pyranrham-nose-(1-6)-β-D-galactopyranose]-5,7,4'-trihydroxyl flavone	III
**13**	4.74	391.1002	-0.63	-	367.1035 $[M-H]^-$	-0.12	MS^2: 353(85.6), 335(100),	368.1107	$C_{17}H_{20}O_9$	5-O-feruloylquinic acid	III

No.	t_R (min)	Positive ESI mode Adduct ions	Mass error (ppm)	MS^n fragment ions	Negative ESI mode Adduct ions	Mass error (ppm)	MS^n fragment ions	Molecular mass	Formula	Identification	Confidence level of identity[a]
		[M+Na]$^+$					**317**(38.5)<br>MS^3: 179(100)				
**14**	4.81	449.1081 [M+H]$^+$	-0.58	-	447.0912 [M-H]$^-$<br>895.1896 [2M-H]$^-$	-0.97	MS^2: 419(10.1), 327(24.8), **301**(44.5), 284(100), 257(8.81)<br>MS^3: 179(100), 151(81.2)	448.1006	$C_{21}H_{20}O_{11}$	quercitrin (**3**)	I
**15**	5.18	-	-	-	191.0335 [M-H]$^-$	-0.41	MS^2: **176**(100)<br>MS^3: 148(100), 120(26.7), 104(15.2)	192.0423	$C_{10}H_8O_4$	scopoletin (**4**)	I
**16**	5.22	-	-	-	433.0765 [M-H]$^-$	2.7	MS^2: 313(54.5), **301**(100), 182(11.8)<br>MS^3: 273(100), 257(87.4), 193(41.3), 178(17.2), 165(10.3)	434.0849	$C_{20}H_{18}O_{11}$	quercetin-3-O-α-L-arabinopyranoside	III
**17**	5.53	415.3940 [M+H]$^+$	-1.34	MS^2: **397**(89.2), 273(100), 233(13.5)<br>MS^3: 255(100), 215(35.1)	-	-	-	414.3862	$C_{29}H_{50}O$	β-sitosterol (**5**)	I
**18**	5.77	-	-	-	1333.6039 [M-H]$^-$	2.34	MS^2: 1249(100), 1189(51.4), 797(86.1)	1334.6143	$C_{63}H_{98}O_{30}$	tubeimoside Ⅱ	III
**19**	5.80	-	-	-	785.4691 [M-H]$^-$	1.17	MS^2: 653(80.6), 635(100), 553(27.5), 535(35.2), 491(70.7), 391(67.8), 343(40.4)	786.4766	$C_{41}H_{70}O_{14}$	tubeimoside Ⅳ	III
**20**	5.80	411.3610 [M+H]$^+$	2.78	MS^2: **393**(81.3), 271(100), 233(26.8)<br>MS^3: 253(100), 215(65.2)	-	-	-	410.3549	$C_{29}H_{46}O$	stigmasta-7,16,25-triene-3-ol	III
**21**	5.82	787.4815 [M+H]$^+$	2.96	-	785.4689 [M-H]$^-$	1.14	MS^2: 653(80.6), 635(100),	786.4766	$C_{41}H_{70}O_{14}$	actinostemmoside F	III

No.	t_R (min)	Positive ESI mode			Negative ESI mode			Molecular mass	Formula	Identification	Confidence level of identity[a]
		Adduct ions	Mass error (ppm)	MS^n fragment ions	Adduct ions	Mass error (ppm)	MS^n fragment ions				
							553(27.5), 535(35.2), 491(70.7), 391(67.8), 343(40.4)				
**22**	6.00	787.4822 [M+H]$^+$	2.07	-	785.4689 [M-H]$^-$	1.14	MS^2: 653(80.6), 635(100), 553(27.5), 535(35.2), 491(70.7), 391(67.8), 343(40.4)	786.4766	$C_{41}H_{70}O_{14}$	7β,18,20,26-tetrahydroxy-(20S)-dammar-24E-en-3-O-α-L-(3-acetyl)arabinopyranosyl-(1→2)-β-D-glucopyranoside	III
**23**	6.05	411.3614 [M+H]$^+$	1.81	MS^2: **393**(100), 273(47.5), 233(40.1)<br>MS^3: 255(100), 215(18.2)	-	-	-	410.3549	$C_{29}H_{46}O$	stigmasta-7,22,25-triene-3-ol	III
**24**	6.10	-	-	-	827.4776 [M-H]$^-$	2.71	MS^2: 827(56.1), 679(100), 621(12.6) **473**(87.2)<br>MS^3: 455(100)	828.4871	$C_{43}H_{72}O_{15}$	7β,18,20,26-tetrahydroxy-(20S)-dammar-24E-en-3-O-α-L-arabinopyranosyl-(1→2)-β-D-(6-acetyl)-glucopyranoside	III
**25**	7.01	1349.6392 [M+H]$^+$	-1.49	-	1347.6194 [M-H]$^-$	2.42	MS^2: **1263**(100)<br>MS^3: 1221(93.6), 1203(100), 1131(18.1) 811(14.2)	1348.6299	$C_{64}H_{100}O_{30}$	lobatoside D	III
**26**	7.26	-	-	-	1363.6154 [M-H]$^-$	-1.81	MS^2: 1279(100), 1219(5.93), 1147(1.0), 1057(5.6), 827(9.63)	1364.6249	$C_{64}H_{100}O_{31}$	tubeimoside Ⅲ	III
**27**	7.39	-	-	-	1317.6083 [M-H]$^-$	2.88	MS^2: 1233(100), 1173(47.3), 781(38.5), 649(11.3)	1318.6194	$C_{63}H_{98}O_{29}$	tubeimoside Ⅰ (**6**)	I

No.	t_R (min)	Positive ESI mode Adduct ions	Mass error (ppm)	MS^n fragment ions	Negative ESI mode Adduct ions	Mass error (ppm)	MS^n fragment ions	Molecular mass	Formula	Identification	Confidence level of identity[a]
**28**	7.44	1217.5969	-1.06	-	1215.5778 [M-H]$^-$	2.14	MS^2: 1041(66.2), **749**(100), MS^3: 587(100), 457(19.3)	1216.5877	$C_{59}H_{92}O_{26}$	lobatoside B	III
**29**	7.51	-	-	-	1231.5735 [M-H]$^-$	1.48	MS^2: 1189(7.9), 1159(13.6) 1147(100), 1105(5.6), 881(9.1), 821(17.0)	1232.5826	$C_{59}H_{92}O_{27}$	dexylosyltubeimoside Ⅲ	III
**30**	7.52	-	-	-	1185.5667 [M-H]$^-$	2.65	MS^2: 1101(100), 1059(14.3), 1041(10.1), 1023(1.2)	1186.5771	$C_{58}H_{90}O_{25}$	lobatoside C	III
**31**	7.67	1217.5915 [M+H]$^+$	2.84		1215.5773 [M-H]$^-$	2.56	MS^2: 1041(66.8), **749(100)** MS^3: 587(100), 457(23.1)	1216.5877	$C_{59}H_{92}O_{26}$	lobatoside G	III
**32**	7.92	-	-	-	1347.6189 [M-H]$^-$	2.79	MS^2: **1263**(100) MS^3: 1221(93.6), 1203(100), 1131((18.1), 811(14.2)	1348.6299	$C_{64}H_{100}O_{30}$	tubeimoside Ⅴ	III
**33**	8.42	1379.6439 [M+H]$^+$	2.81		1377.6297 [M-H]$^-$	2.56	MS^2: **1217**(100) MS^3: 895(100), 733(25.8),455(17.2), 137(11.3)	1378.6405	$C_{65}H_{102}O_{31}$	lobatoside F	III
**34**	8.89	817.4951 [M+H]$^+$	-0.86	-	815.4788 [M-H]$^-$	1.88	MS^2: 797(1.3) **769**(100), 635(1.0), 559(1.6) MS^3: 701(33.2), 637(100), 619(32.8)	816.4871	$C_{42}H_{72}O_{15}$	actinostemmoside H	III
**35**	9.71	771.4891 [M+H]$^+$	-0.23	-	769.4722 [M-H]$^-$	2.81	MS^2: 637(62.2), 619(100), 589(7.4), 571(9.3),	770.4816	$C_{41}H_{70}O_{13}$	7β,20,26-trihydroxy-(20S)-dammar-24E-en-3-O-α-L-	III

No.	t_R (min)	Positive ESI mode Adduct ions	Mass error (ppm)	MSⁿ fragment ions	Negative ESI mode Adduct ions	Mass error (ppm)	MSⁿ fragment ions	Molecular mass	Formula	Identification	Confidence level of identity[a]
							475(67.9), 427(8.0), 343(24.4)			arabinopyranosyl-(1→2)-β-D-glucopyranoside	
**36**	9.76	-	-	-	781.4365 [M-H]$^-$	1.89	MS^2: 649 469	782.4453	$C_{41}H_{66}O_{14}$	lobatoside A	III
**37**	10.87	-	-	-	811.4829 [M-H]$^-$ 1623.9679 [2M-H]$^-$	2.50	MS^2: 769(100), 751(4.6) 619(11.2), 571(6.7)	812.4922	$C_{43}H_{72}O_{14}$	7β,20,26-trihydroxy-(20S)-dammar-24E-en-3-O-α-L-(3-acetyl)arabinopyranosyl-(1→2)-β-D-glucopyranoside	III
**38**	11.68	-	-	-	799.4831 [M-H]$^-$	2.29	MS^2: 637(33.7), **475**(100), 457(14.7) MS^3: 439(100)	800.4922	$C_{42}H_{72}O_{14}$	actinostemmoside E	III
**39**	13.19	271.0589 [M+H]$^+$	-1.230	-	269.0457 [M-H]$^-$	0.77	MS^2: 241(32.2), 225(100), 197(3.98)	270.0528	$C_{15}H_{10}O_5$	emodin (**7**)	I
**40**	13.67	-	-	-	559.3260 [M-H]$^-$	2.94	MS^2: 541(26.9), 499(38.9), **481**(100) MS^3: 463(46.3), 439(27.0), 301(100), 283(24.5)	560.3349	$C_{32}H_{48}O_8$	23,24-dihydroisocucurbitacin B	III
**41**	13.76	285.0746 [M+H]$^+$	-0.92		283.0605 [M-H]$^-$	2.43	MS^2: 269 241 225 197	284.0685	$C_{16}H_{12}O_5$	emodinmonomethylether	III
**42**	14.48	-	-	-	559.3266 [M-H]$^-$	1.86	MS^2: 541(26.9), 499(38.9), **481**(100) MS^3: 463(46.3), 439(27.0), 301(100), 283(24.5)	560.3349	$C_{32}H_{48}O_8$	cucurbitacin E	III
**43**	15.03	699.3602 [M+H]$^+$	-1.43	MS^2: 615(54.2), 575(15.6), **521**(100), 437(34.8), 397(19.7) MS^3: 357(100), 273(21.5), 233(17.6)	697.3450 [M-H]$^-$	-1.32	-	698.3514	$C_{35}H_{54}O_{14}$	uzarigenin-3-β-sophoroside	III
**44**	15.27	581.3100	-2.60	-	557.3102 [M-H]$^-$	2.85	MS^2: 539(100), 515(30.8),	558.3193	$C_{32}H_{46}O_8$	cucurbitacin B (**8**)	I

No.	t_R (min)	Positive ESI mode			Negative ESI mode			Molecular mass	Formula	Identification	Confidence level of identity[a]
		Adduct ions	Mass error (ppm)	MS^n fragment ions	Adduct ions	Mass error (ppm)	MS^n fragment ions				
		[M+Na]$^+$					497(90.1) 479(10.2)				
**45**	16.57	701.3750 [M+H]$^+$	-0.67	MS^2: 701(51.2), **683**(89.3), 523(100), 335(35.9), 317(24.6), 277(12.3) MS^3: 665(100), 487(32.6), 299(33.7), 259(19.4)	699.3602 [M-H]$^-$	-0.67	-	700.3670	$C_{35}H_{56}O_{14}$	sileneoside H	III
**46**	16.94	577.4455 [M+H]$^+$	1.33	MS^2: 435(63.5), **397**(100), MS^3: 255(100),215(29.7)	575.4308 [M-H]$^-$	1.59	-	576.4390	$C_{35}H_{60}O_6$	daucosterol	III
**47**	17.56	537.3066 [M+H]$^+$	-0.75	MS^2: 453(56.3), 413(33.2), 395(21.4), **373**(100) MS^3: 289(100), 249(45.8), 231(39.7)	535.2900 [M-H]$^-$	2.35	-	536.2985	$C_{29}H_{44}O_9$	frugoside	III
**48**	17.96	573.4139 [M+H]$^+$	1.86	MS^2: 435(100), **393**(86.3) MS^3: 255(100), 215(37.2)	571.3995 [M-H]$^-$	1.60	-	572.4077	$C_{35}H_{56}O_6$	stigmasta-7,22,25-triene-3-O-β-D-glucopyranoside	III
**49**	18.77	-	-	-	1191.5806 [M-H]$^-$	0.77	MS^2: 1059(71.4), 897(36.7), 765(15.1) **487**(100) MS^3: 469(100)	1192.5877	$C_{57}H_{92}O_{26}$	3-O-α-L-arabinopyranosyl(1→2)-β-D-glucopyranosyl-bayogenin-28-O-β-D-xylopyranosyl(1→3)-α-L-rhamnopyranosyl(1→2)-α-L-arabinopyranoside	III
**50**	19.32	539.3218 [M+H]$^+$	-0.10	MS^2: **521**(93.1), 335(100), 295(25.9), 277(63.3) MS^3: 503(100), 285(72.3), 245(61.3), 227(27.1)	537.3055 [M-H]$^-$	2.62	-	538.3142	$C_{29}H_{46}O_9$	integristerone A-25-acetate	III
**51**	19.96	493.3160 [M+H]$^+$	0.48	MS^2: **475**(100), 319(89.8), 279(54.7), 261(39.1) MS^3: 457(100), 269(39.3),	-	-	-	492.3087	$C_{28}H_{44}O_7$	24(28)-dehydromakisterone A	III

No.	t_R (min)	Positive ESI mode Adduct ions	Mass error (ppm)	MS^n fragment ions	Negative ESI mode Adduct ions	Mass error (ppm)	MS^n fragment ions	Molecular mass	Formula	Identification	Confidence level of identity[a]
				229(38.4), 211(15.2)							
**52**	20.99	279.1595 [M+H]$^+$	-1.24	MS^2: 279(100), 223(59.8), 167(65.3)	-	-	-	278.1518	$C_{16}H_{22}O_4$	di-butyl phthalate	III
**53**	22.96	653.6215 [M+H]$^+$	2.46	MS^2: 511(78.2), **397**(100)<br>MS^3: 255(100), 215(67.2)	-	-	-	652.6158	$C_{45}H_{80}O_2$	β-sitosterol palmitate	III
**54**	23.67	811.6425 [M+H]$^+$	2.63	MS^2: 573(100), 435(56.1), **393**(79.2)<br>MS^3: 255(100), 215(63.7)	809.6290 [M-H]$^-$	1.33	-	810.6374	$C_{51}H_{86}O_7$	stigmasta-7,22,25-triene-3-O-β-D-(6′-palmitoyl) glucopyranoside	III
**55**	24.24	691.6377 [M+H]$^+$	1.53	MS^2: 553(59.9), **393**(100)<br>MS^3: 255(100), 215(55.4)	-	-	-	690.6315	$C_{48}H_{82}O_2$	stigmasta-7,22,25-triene-3-O-nonadecanoic acid ester	III
**56**	24.91	282.2799 [M+H]$^+$<br>563.5530 [2M+H]$^+$	-2.69	MS^2: 237(100)	280.2640 [M-H]$^-$	2.10	-	281.2719	$C_{18}H_{35}NO$	9-octadecenamide	III
**57**	25.25	314.1379 [M+H]$^+$	2.50	-	312.1249 [M-H]$^-$	-2.46	MS^2: 193 136	313.1314	$C_{18}H_{19}NO_4$	(E)-N-hydroxy phenylethyl-3-(4-hy-droxy-3-methoxy phenyl) acrylamide	III
**58**	26.51	-	-	-	255.2328 [M-H]$^-$	0.66	MS^2: 209(100)	256.2402	$C_{16}H_{32}O_2$	hexadecanoic acid	III
**59**	27.17	595.3974 [M+Na]$^+$	-0.91	MS^2: 435(70.8), 395(100), **393**(94.2)<br>MS^3: 255(100), 215(36.9)	-	-	-	572.4077	$C_{35}H_{56}O_6$	(3β,22E)-stigmasta-7,22,25-trien-3-yl-β-D-glucopyranoside	III
**60**	27.62	327.1958 [M+H]$^+$	-1.01	MS^2: 255(100)<br>MS^3: 215(100)	325.1818 [M-H]$^-$	-2.66	-	326.1882	$C_{21}H_{26}O_3$	3-oxo-androsta-1,4-dien-17a′-spiro-2′-3′-oxo-oxetane	III

[a] The confidence level of identity of all the identified components were determined following the four levels defined by the Metabolomics Standards Initiative. Level Ⅰ: confidently identified compounds; Level Ⅱ: Putatively annotated compounds; Level Ⅲ: putatively annotated compound classes; Level Ⅳ: unknown compounds.

[b] The bold m/z values and bracketed relative peak intensities showed the targeted MS^2 fragment ions for further MS^3 fragmentation.

[c] The bracketed bold figures showed the serial number of corresponding reference compounds.

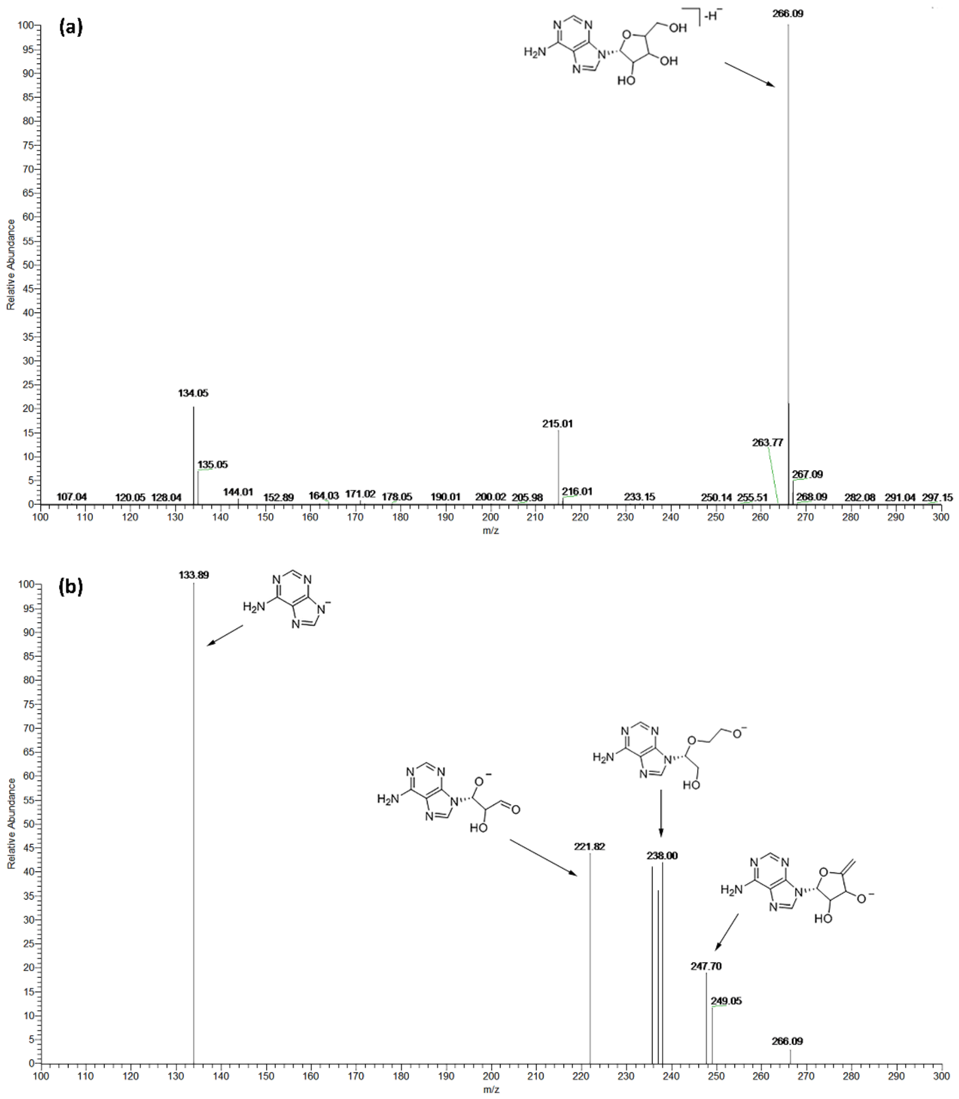

Figure S1. MS^n spectra and proposed fragment ions of adenosine (**1**) in negative ion mode: (**a**) MS spectrum; (**b**) MS^2 spectrum (precursor ion was m/z 266).

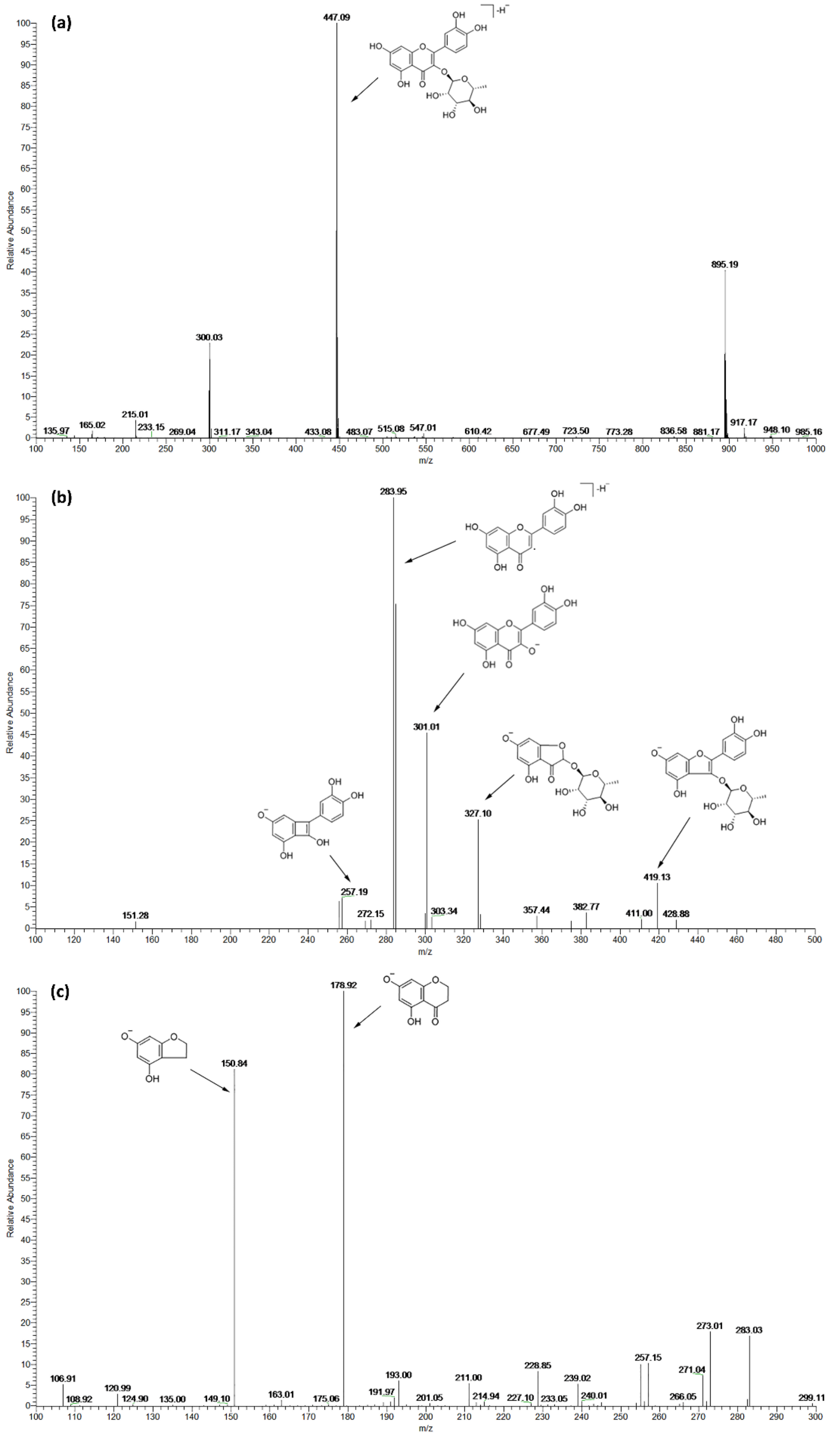

Figure S2. MS^n spectra and proposed fragment ions of quercitrin (**3**) in negative ion mode: (**a**) MS spectrum; (**b**) MS^2 spectrum (precursor ion was m/z 447); (**c**) MS^3 spectrum (precursor ion was m/z 301).

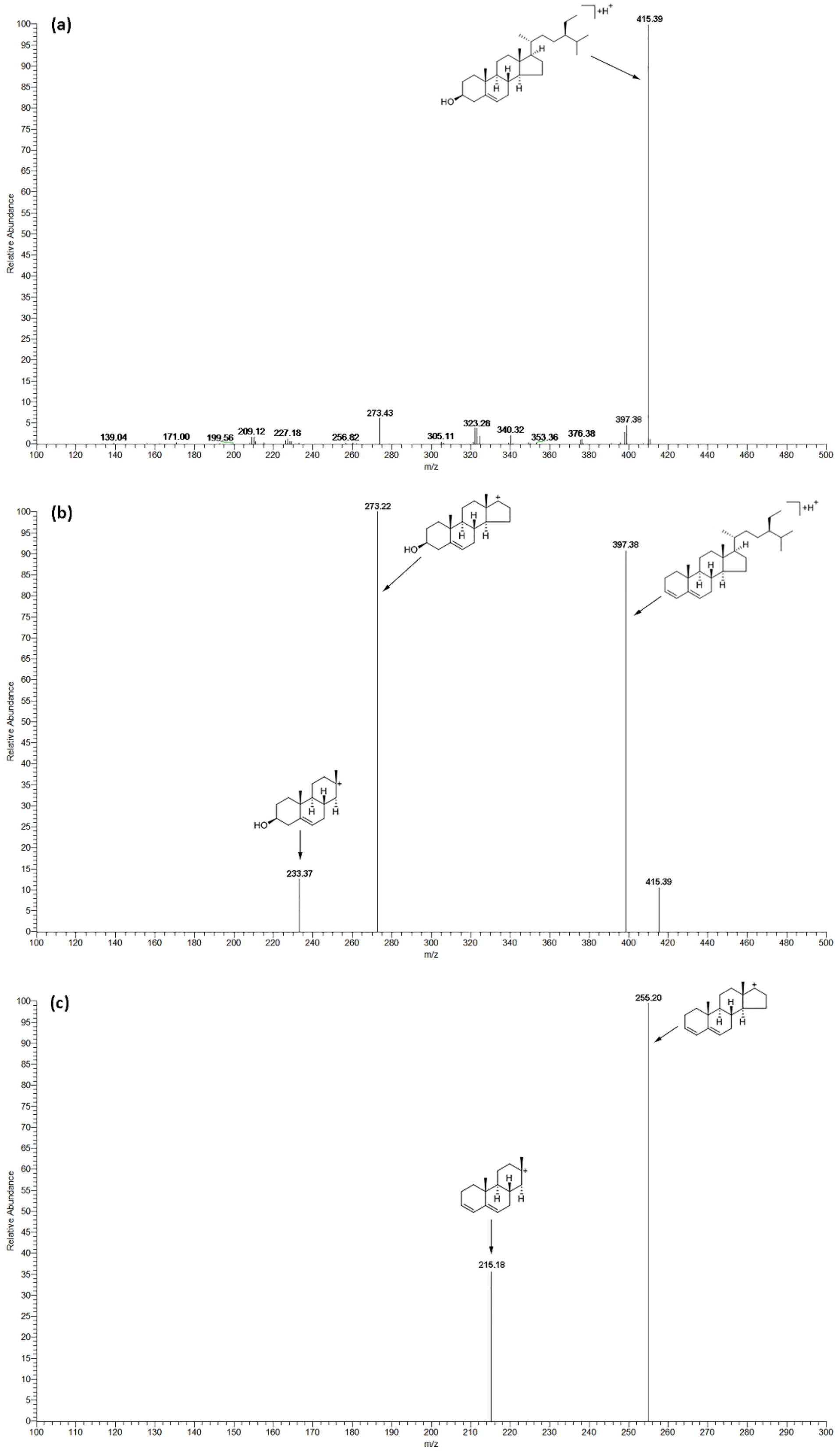

Figure S3. MS^n spectra and proposed fragment ions of β-sitosterol (**5**) in positive ion mode: (**a**) MS spectrum; (**b**) MS^2 spectrum (precursor ion was m/z 415); (**c**) MS^3 spectrum (precursor ion was m/z 397).

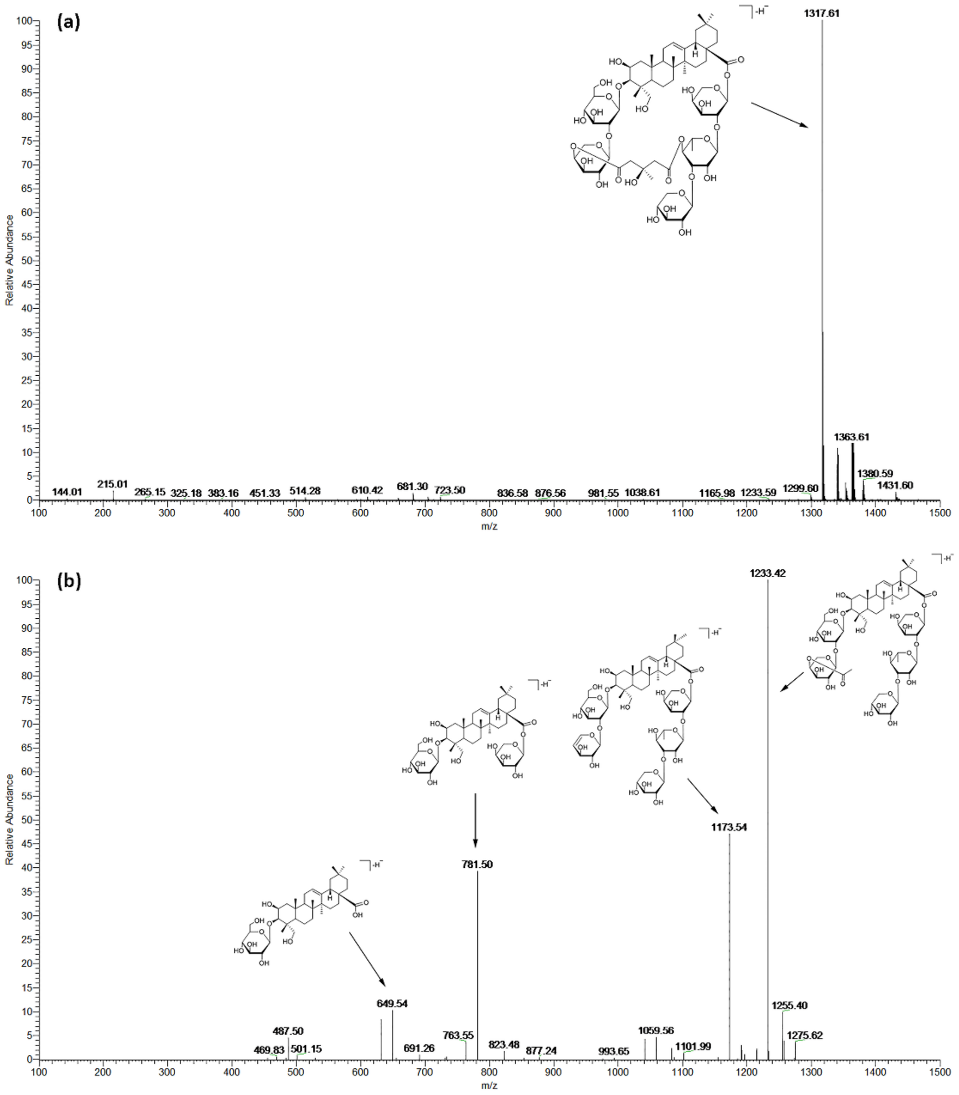

Figure S4. MS^n spectra and proposed fragment ions of tubeimoside Ⅰ (**6**) in negative ion mode: (**a**) MS spectrum; (**b**) MS^2 spectrum (precursor ion was m/z 1317).

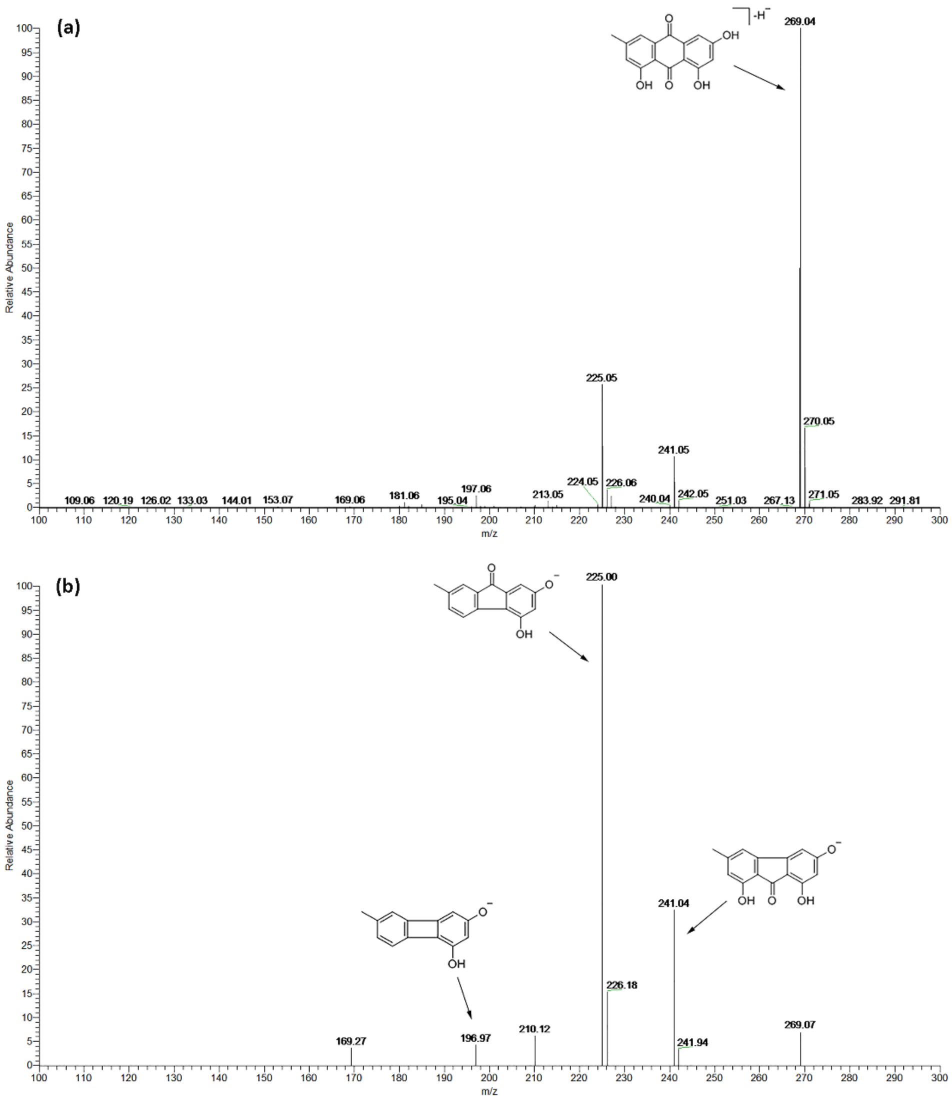

Figure S5. MS^n spectra and proposed fragment ions of emodin (**7**) in negative ion mode: (**a**) MS spectrum; (**b**) MS^2 spectrum (precursor ion was m/z 269).

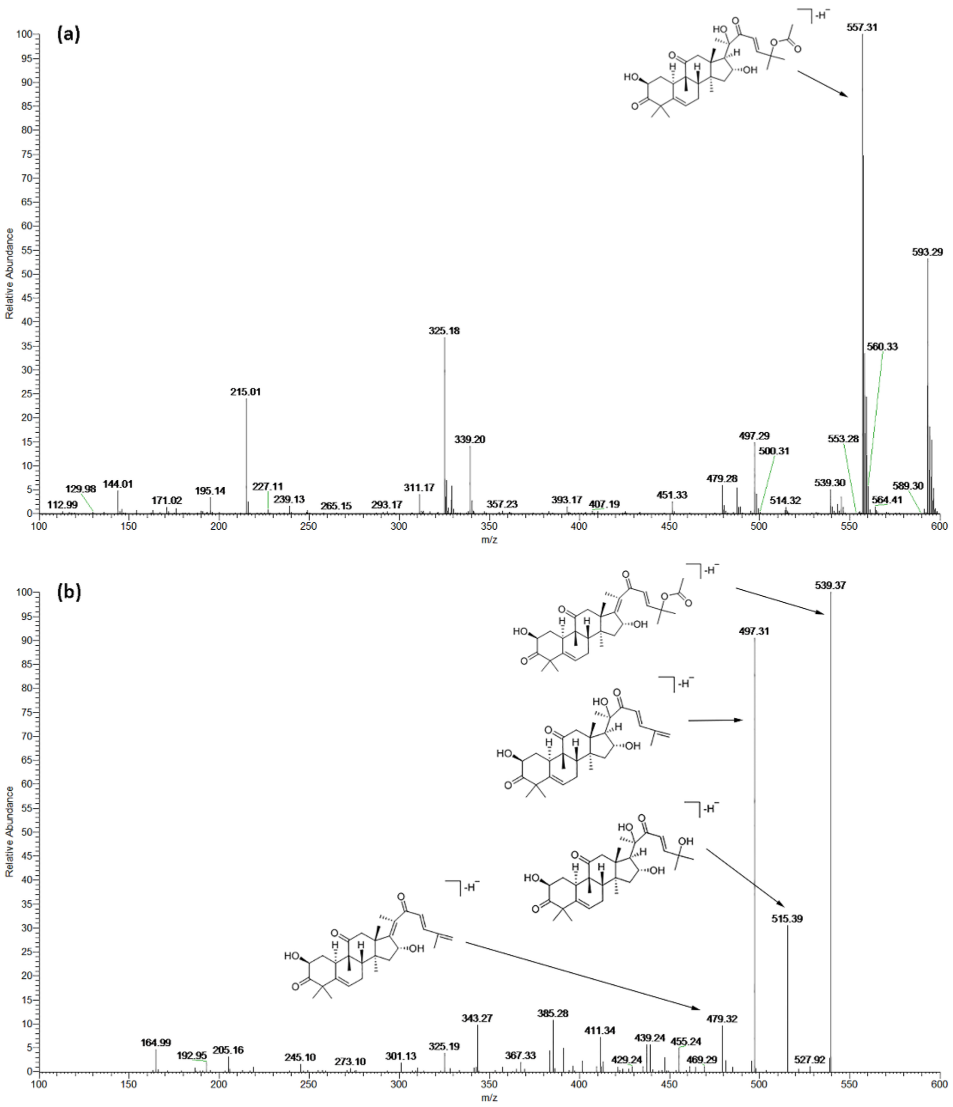

Figure S6. MS^n spectra and proposed fragment ions of cucurbitacin B (**8**) in negative ion mode: (**a**) MS spectrum; (**b**) MS^2 spectrum (precursor ion was m/z 557).

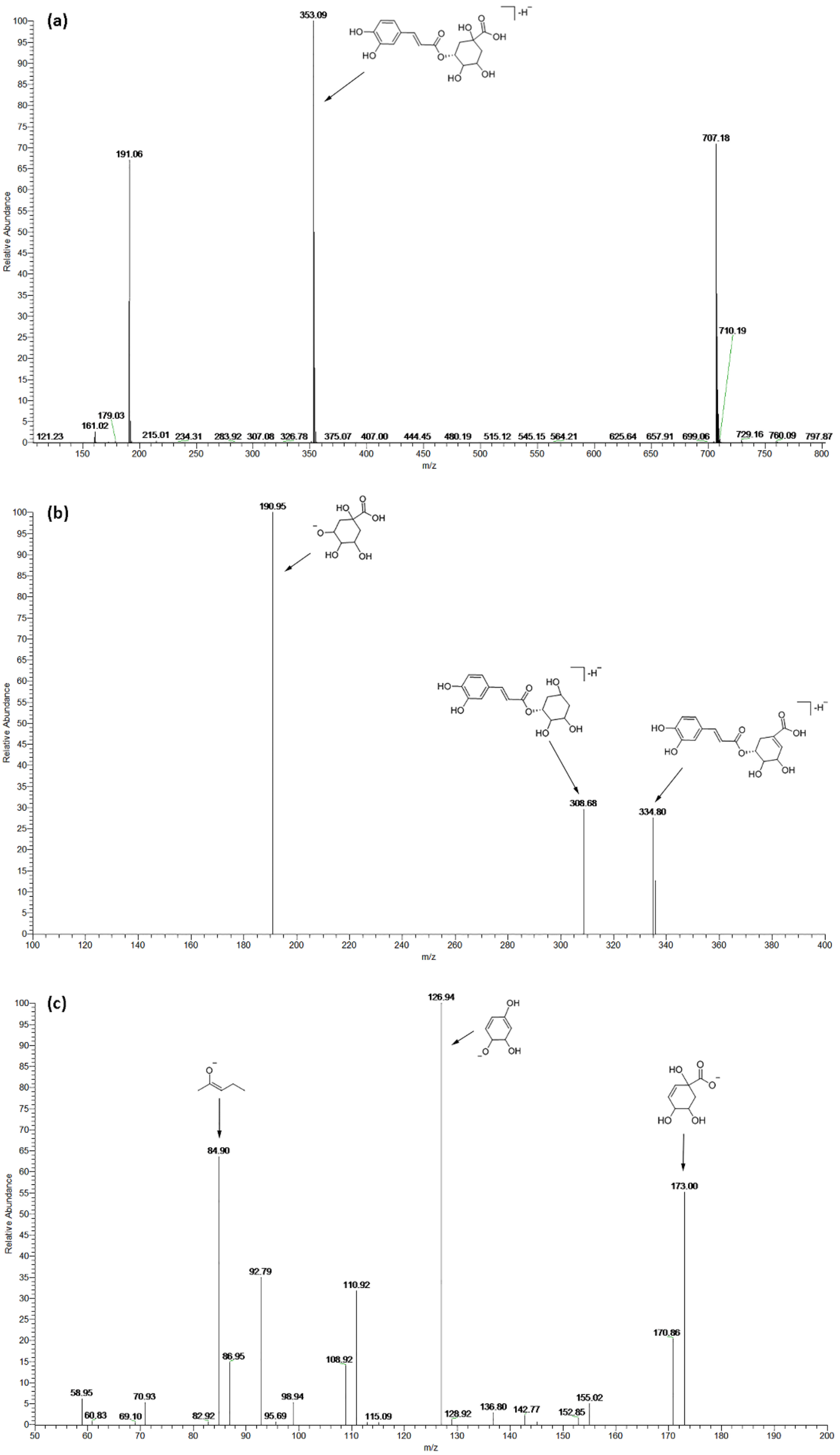

Figure S7. MS^n spectra and proposed fragment ions of chlorogenic acid (**2**) in negative ion mode: (**a**) MS spectrum; (**b**) MS^2 spectrum (precursor ion was m/z 353); (**c**) MS^3 spectrum (precursor ion was m/z 191).

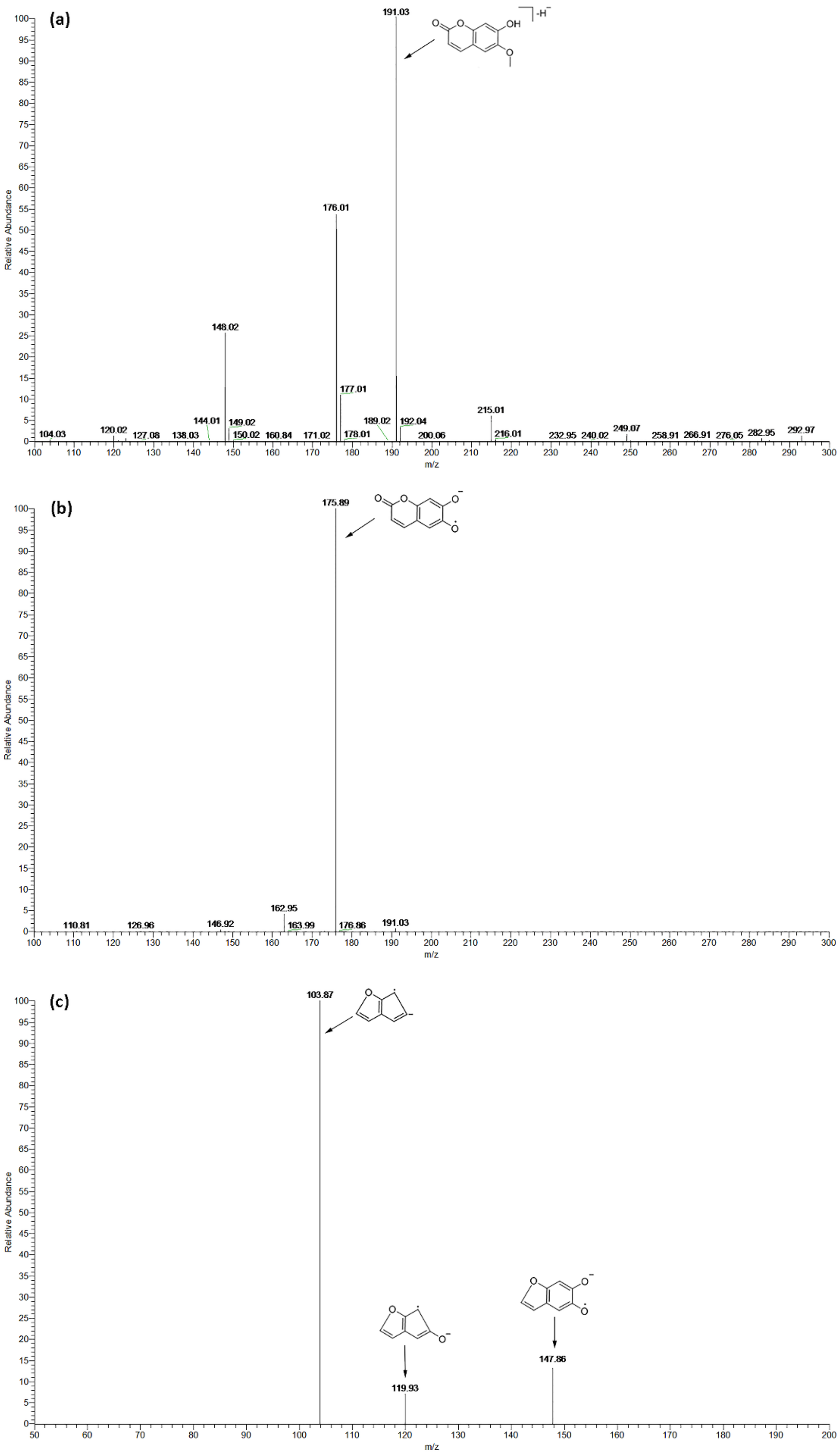

Figure S8. MS^n spectra and proposed fragment ions of scopoletin (**4**) in negative ion mode: (**a**) MS spectrum; (**b**) MS^2 spectrum (precursor ion was m/z 191); (**c**) MS^3 spectrum (precursor ion was m/z 176).
